# 

**Published:** 1895-01-05

**Authors:** 


CONTENTS.

**Annus Medicus, 1894** - 233

**Annotations.**—Cholera Innoculation: More Evidence—"Men's Children"—The Domestic Filter - 237

**Medical Progress and Hospital Clinics:**

Diphtheritic Paralysis in Children - 239

PROGRESS IN SURGERY—Diseases of the Liver—General Surgery 240

TOXICOLOGICAL NOTE — Poisoning from Subnitrate of Bismuth - 244

**Notes and News** - 238

**The Institutional Workshop:**

THE FINANCIAL STRENGTH OF OUR VOLUNTARY CHARITIES - 245

PRACTICAL DEPARTMENTS.—Children's Cots - 247

CONSTRUCTION NOTES - 248

**Editor's Letter-Box.**—The Radcliffe Infirmary, Oxford - 248

**Scraps and Gleanings** - 248

**The Book World of Medicine and Science** - 249

EXTRA SUPPLEMENT.—THE NURSING MIRROR.

**News from the Nursing World.**—Organised by Princess Beatrice—The Empress Frederick—Westminster Hospital—The Caznove Prizes—Awaiting the Decision—Past and Present—A Good Year of Holly—Midwives—Private Nurses at Southampton—Isolation for Suspicious Cases—Caged Children—A Nurses' Home Wanted—Queen's Nurse at Arbroath—A New York Club—Short Items - ci

**The Dietetic Management of Infancy.** IV.—Atrophy cii

**Wants and Workers** - ciii

**The American Woman at Home.** V.—Her Lovers - civ

**Appointments** - civ

**Everybody's Opinion**—An Appropriate Thank-offering - cvi

**Presentations** - cvi

**Minor Appointment** - civ

**Christmas in Germany** - cvi

**Entertainments** - cvii

